# Hippocampal GFAP in aging: Associations with AD and LATE‐NC pathologies and cognitive decline in older adults

**DOI:** 10.1002/alz.71613

**Published:** 2026-06-17

**Authors:** Sonal Agrawal, Lei Yu, Sue E. Leurgans, Jialiang Li, Alifiya Kapasi, Puja Agarwal, Jeffrey L. Dage, Lisa L. Barnes, David A. Bennett, Patricia Boyle, Julie A. Schneider

**Affiliations:** ^1^ Rush Alzheimer's Disease Center Rush University Medical Center Chicago Illinois USA; ^2^ Department of Pathology Rush University Medical Center Chicago Illinois USA; ^3^ Department of Neurological Sciences Rush University Medical Center Chicago Illinois USA; ^4^ Department of Internal Medicine Rush University Medical Center Chicago Illinois USA; ^5^ Department of Neurology Indiana University School of Medicine Indianapolis Indiana USA; ^6^ Department of Medical and Molecular Genetics Indiana University School of Medicine Indianapolis Indiana USA; ^7^ Department of Psychiatry and Behavioral Sciences Rush University Medical Center Chicago Illinois USA

**Keywords:** Alzheimer's disease, Astrocytes, cognition, dementia, GFAP, LATE‐NC, tangles, TDP‐43, digital pathology

## Abstract

**INTRODUCTION:**

Plasma glial fibrillary acidic protein (GFAP) is an emerging biomarker for Alzheimer's disease (AD) progression in clinical studies, yet the role of brain GFAP in AD/AD‐related dementias (ADRD) pathologies and cognitive decline remains unclear.

**METHODS:**

GFAP burden from CA1‐subiculum of the hippocampus were quantified. Regression and mixed‐effect models, adjusting for demographics and other brain pathologies examined associations between hippocampal GFAP and AD/ADRD pathologies and separately with Alzheimer's dementia and cognitive decline.

**RESULTS:**

Limbic‐predominant age‐related TDP‐43 encephalopathy neuropathologic changes (LATE‐NC), hippocampal sclerosis of aging (HS‐A), and neurofibrillary tangle density (but not amyloid‐beta) were associated with GFAP burden. Hippocampal GFAP was associated with increased odds of Alzheimer's dementia and faster decline in global cognition, episodic memory, semantic memory, and perceptual speed. LATE‐NC and tangles explained some but not all the association between hippocampal GFAP and cognitive decline.

**DISCUSSION:**

GFAP burden in the hippocampus is related to LATE‐NC and tangles but may also be an independent contributor to cognitive decline.

## Highlights

- Hippocampal glial fibrillary acidic protein (GFAP) is strongly associated with limbic‐predominant age‐related TDP‐43 encephalopathy neuropathologic changes (LATE‐NC), hippocampal sclerosis of aging (HS‐A), and tangles, but not with amyloid‐β and cerebrovascular pathologies.
- Increased hippocampal GFAP burden independently predicts Alzheimer's dementia and cognitive decline.
- LATE‐NC and tangles account for much, but not all, of the association between hippocampal GFAP and cognitive decline.

## 1 BACKGROUND

Astrocytes, characterized by their star‐shaped morphology, are the most abundant glial cells in the central nervous system (CNS), constituting approximately 40%–60% of the total glial population.^1^ These cells play crucial roles in supporting neuronal metabolism, synaptic activity, and regulating the blood‐brain barrier.^2^, ^3^, ^4^, ^5^ However, astrocytes can transform into reactive astrocytes, marked by increased expression of glial fibrillary acidic protein (GFAP), and thereby contribute to neuroinflammation and neurodegeneration in several neurodegenerative diseases, such as Alzheimer's disease (AD), Parkinson's disease, and stroke.^6^, ^7^ Specifically in AD, studies have shown that reactive astrocytes secrete proinflammatory cytokines, facilitate amyloid‐β accumulation, localize near neuritic amyloid‐β plaques, and contribute to neuronal damage.^8^, ^9^, ^10^ Interestingly, genome‐wide association studies have identified risk genes for AD that are highly expressed by astrocytes including apolipoprotein E (APOE), suggesting that molecular pathways in astrocytes may contribute to AD risk.^11^

The aging brain often harbors multiple pathologies other than AD, many of which contribute to cognitive impairment and lower the threshold for Alzheimer's dementia.^12^, ^13^, ^14^ Limbic‐predominant age‐related TDP‐43 encephalopathy neuropathologic change (LATE‐NC) is recognized as an important pathology in older adults that shows detrimental effect in late‐life cognitive function particularly in memory function.^15^, ^16^ In a recent study, we demonstrated the importance of microglia in relation to LATE‐NC and cognitive decline.^17^ However, the involvement of astrocytosis in LATE‐NC pathophysiological processes remains unclear, especially independent from AD, as both pathologies tend to co‐exist and accumulate in the hippocampus. Despite the substantial role of LATE‐NC in Alzheimer's dementia and aging, the involvement of astrocytosis in LATE‐NC pathophysiological processes remains unclear, especially independent from AD, as both pathologies tend to co‐exist and accumulate in the hippocampus. Moreover, the associations of astrocytosis with Alzheimer's dementia and cognitive decline, independent of age‐related brain pathologies, have not been fully elucidated. Investigating these relationships could provide valuable insights into their role in Alzheimer's disease and Alzheimer's disease‐related dementias (AD/ADRD).

To address this gap in the literature, we utilized immunohistochemistry along with digital image analysis to quantify GFAP burden as a marker of reactive astrocytes in the hippocampus, a limbic region critical for memory formation and particularly vulnerable to AD and LATE‐NC. Clinical and neuropathological *postmortem* data came from 407 decedents enrolled in the Religious Orders Study, the Rush Memory and Aging Project, and the Minority Aging Research Study. We first examined the association of hippocampal GFAP burden with LATE‐NC, AD, and other brain pathologies. Subsequently, we examined the associations of hippocampal GFAP burden with Alzheimer's dementia and cognitive decline after controlling demographics and brain pathologies and assessed how much of the association of hippocampal GFAP burden with cognitive decline was relatively independent versus attributable to other brain pathologies.

## 2 METHODS

### 2.1 Study participants

Participants were selected from the three ongoing clinicopathologic cohort studies of aging: the Religious Orders Study, the Rush Memory and Aging Project, and the Minority Aging Research Study^18^, ^19^; here after referred to as ROS/MAP/MARS. Participants in all these studies enrolled without known dementia and were followed longitudinally for annual cognitive assessment. Based on specific enrollment criteria for each study, brain donation was mandatory for ROS and MAP participants, but optional for MARS participants. Each study research protocol was approved by a Rush University Medical Center institutional review board.

The inclusion criteria of this study required completion of baseline evaluation and at least one follow‐up cognitive assessment, as well as neuropathologic data on hippocampal GFAP and other brain pathologies. Participants with pathologic diagnosis of frontotemporal lobar degeneration were excluded from the study. Based on the inclusion and exclusion criteria, a total of 407 participants were eligible at the time when the data were extracted for analysis in March 2025. A flow chart showing the reasons for inclusion and exclusion are shown in Figure S1.

### 2.2 Assessment of cognitive function and Alzheimer's dementia

All participants underwent annual uniform structured clinical examinations that included neuropsychological testing, medical history, and neurologic evaluation. Annual clinical assessments were performed by clinicians who were blinded to prior assessments and in‐vivo neuroimaging evaluation. Nineteen neuropsychological tests were employed to compute quantitative summaries of cognitive function both globally and for five specific cognitive domains: episodic memory, semantic memory, working memory, perceptual speed, and visuospatial ability. These composite measures were created by averaging standardized scores obtained from individual tests.^20^, ^21^

The clinical diagnosis of Alzheimer's dementia was established using the criteria set by the joint working group of the National Institute of Neurological and Communicative Disorders and Stroke and the Alzheimer's Disease and Related Disorders Association.^22^ The diagnosis used the information from a structured neurological examination, medical history, and cognitive performance assessments and reviewed by the experienced clinician after death. An Alzheimer's dementia diagnosis required a history of cognitive decline with episodic memory impairment and impairment in at least one additional cognitive domain. Mild cognitive impairment (MCI) indicated to those assessed as cognitively impaired by the neuropsychologist but determined to not have dementia by the clinician.^23^

### 2.3 Neuropathologic evaluations

A standard protocol was used for brain removal which is performed at a median of 8 (interquartile range: 6–12) h after death.^24^ One hemisphere with no gross pathology was frozen, and the other hemisphere with less mechanical damage or having gross pathology was fixed for at least three days using 4% paraformaldehyde. A standard gross and microscopic neuropathological examinations were then carried out blinded to clinical and ex‐vivo/in‐vivo neuroimaging evaluations, which included assessments of *postmortem* indices describing the GFAP burden on the hippocampus, and the pathologic diagnoses of AD, LATE‐NC, and other pathologies, consistent with earlier established previously published procedures.^25^, ^26^

#### 2.3.1 Hippocampal GFAP burden

GFAP data collection on the hippocampus began in 2018 and was collected prospectively in ROSMAPMARS decedents. Data were gathered from the mid‐hippocampus at the level of the lateral geniculate nucleus. Immunohistochemistry using GFAP monoclonal antibody (clone GA5; Leica Biosystems Inc, IL; catalog # GFAP‐GA5‐L‐U; diluted at 1:1000) was applied to 6‐µm paraffin‐embedded tissue sections. To minimize potential batch staining effects, staining was carried out using standardized protocols, including the same antibody lots, staining platforms, antigen retrieval conditions, and detection systems across runs. Positive and negative control tissues were also included in each batch to ensure consistent staining performance. In addition, we examined slide‐to‐slide variation to assess reproducibility across batches. We selected two consecutive slides of the mid‐hippocampus (*N* = 50) and stained with GFAP, scanned, annotated, and analyzed separately in two batches. The intraclass correlation coefficient for hippocampal GFAP burden is high such that 88% of variability for GFAP is due to person‐to‐person variation, suggesting good reproducibility and minimal slide‐to‐slide variation.

**RESEARCH IN CONTEXT**

1. **Systematic review**: The authors reviewed the literature using traditional (e.g., PubMed) sources and meeting abstracts and presentations about the association of brain astrocytes with Alzheimer's disease and Alzheimer's disease‐related dementias (AD/ADRD) pathologies, Alzheimer's dementia and cognitive decline. The relevant citations are appropriately cited. There are limited *postmortem* studies examining the association of hippocampal glial fibrillary acidic protein (GFAP) with AD vs ADRD brain pathologies particularly with limbic‐predominant age‐related TDP‐43 encephalopathy neuropathologic changes (LATE‐NC), and very little is known regarding the relationship between brain GFAP, neurodegenerative and vascular pathologies, and cognition.
2. **Interpretation**: Our findings demonstrate that higher GFAP burden in the hippocampus specifically associated with LATE‐NC, hippocampal sclerosis of aging (HS‐A), and less so with AD pathologies. Besides the associations of GFAP with brain pathologies, GFAP in the hippocampus is an important predictor of Alzheimer's dementia and contributes to late‐life cognitive decline, above and beyond the presence of common neurodegenerative and cerebrovascular pathologies. Specifically, GFAP burden in the hippocampus was associated with decline in domains of episodic memory, semantic memory, and perceptual speed. Further, findings show that one thirds of the association between GFAP burden in the hippocampus and cognitive decline is mediated through tangles and LATE‐NC, but remaining larger proportions are independent from brain pathologies.
3. **Future directions**: Future understanding of the cellular interactions among astrocytes, other glial cells (including microglia and oligodendrocytes), and neurons will offer valuable insights into regional heterogeneity within the brain. Additionally, further exploration of the relationships between brain GFAP levels, plasma biomarkers, and neuroimaging markers of AD will enhance our understanding of disease progression and help to distinguish AD from other brain pathologies.

**FIGURE 1:**
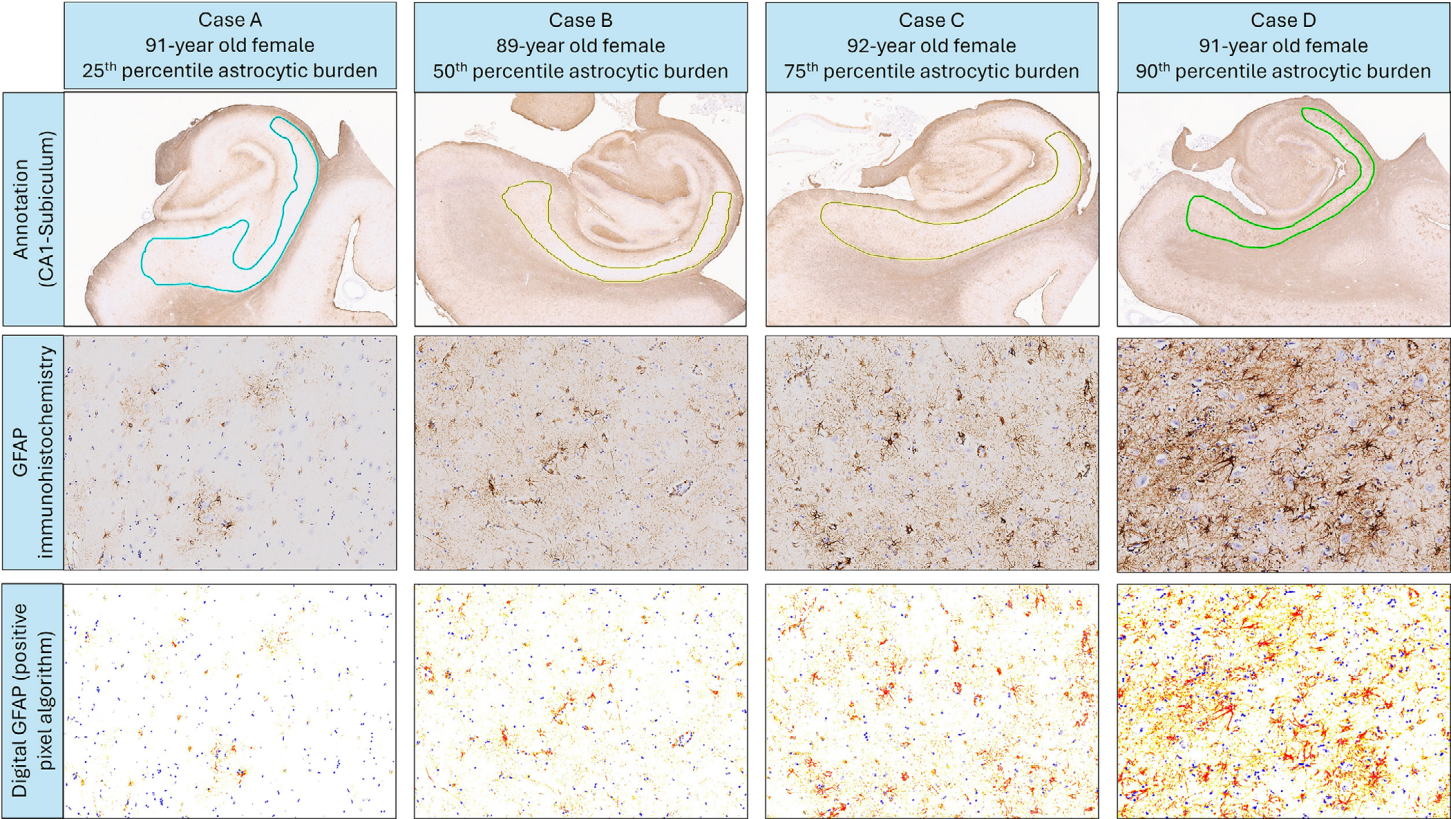
Hippocampal GFAP immunostaining. The upper panel displays the annotated ROI), specifically the CA1–subiculum region of the mid‐hippocampus, collected at the level of the lateral geniculate nucleus, from four participants (GFAP burden levels: 25^th^, 50^th^, 75^th^, 90^th^ percentile). The middle panel presents high‐magnification views from the ROI. The lower panel illustrates hippocampal GFAP burden, identified using the positive pixel algorithm. GFAP, glial fibrillary acidic protein; ROI, region of interest.

After completing internal quality control procedures, stained slides were scanned into the Aperio AT2 scanner console and processed into digital images. To quantify GFAP burden in the hippocampus, a trained rater digitally outlined the regions of interest (CA1 and subiculum) following training with a neuropathology experts (Figure S2). As part of this training, a range of cases, including those with different levels of ADNC or HS‐A, were reviewed to reach consensus on the CA1–subiculum boundaries. This outlining approach has been used previously and extensively published in our previous studies ^17^, ^27^. Then, the Aperio Image Analysis Toolbox Positive Pixel Count algorithm was used to optimize the parameters to specify weak, medium, and strong thresholds for GFAP staining intensity. The hippocampal GFAP fraction (positivity/area outlined) was calculated as the total number of pixels rated as weak (+1, yellow), medium (+2, orange), and strong (+3, red) GFAP staining intensity divided by the area outlined. The resulting fraction (positivity/area outlined) was expressed as a percentage by multiplying 100. We then applied the square root transformation to obtain GFAP score within the region and averaged among the participants to estimate hippocampal GFAP burden. Note that hippocampal GFAP burden calculated for this study includes astrocytes cell bodies together with their processes. Hippocampal GFAP staining and its quantification are presented in Figure 1.

#### 2.3.2 LATE‐NC pathology

Immunohistochemistry employing a monoclonal antibody to phosphorylated TDP‐43 (pS409/410; Biolegend, San Diego, CA; catalog # 829901; diluted at 1:10,000) was used to identify phosphorylated transactive response DNA‐binding protein 43 (TDP‐43) pathology. TDP‐43 was evaluated for eight brain regions, including the amygdala, hippocampus dentate gyrus and CA1‐subiculum, entorhinal cortex, middle temporal gyrus, anterior temporal pole, midfrontal gyrus, and orbitofrontal cortex.^28^ Based on modified LATE‐NC consensus criteria ^26^, four distinct stages were defined: stage 0 (no TDP‐43), stage 1 (TDP‐43 in the amygdala), stage 2 (TDP‐43 in the hippocampus and/or entorhinal cortex, but not the neocortex), and stage 3 (TDP‐43 in the neocortex). For descriptive and analytic analyses, we used the four stages of LATE‐NC. In addition, we created a dichotomous variable by classifying participants with stages 0 and 1 as LATE‐NC (–), and those with stages 2 and 3 as LATE‐NC (+).^29^, ^30^ Note that LATE‐NC positivity was defined with or without the presence of hippocampal sclerosis of aging (HS‐A) in this study. Measure of hippocampal TDP‐43 cytoplasmic inclusions severity was used in path analyses.

#### 2.3.3 Alzheimer's disease pathology

Manual counts of neurofibrillary tangles and neuritic plaques were conducted in areas of greatest density by using a modified Bielschowsky stain from five brain regions including three neocortical (middle temporal, mid frontal, inferior parietal) and two limbic regions (entorhinal cortex and hippocampus CA1) to determine Braak Neurofibrillary Tangle (NFT) staging (0–VI) and Consortium to Establish a Registry for Alzheimer's Disease (CERAD) scores.^31^ For supporting analyses, the counts of neurofibrillary tangles and of neuritic plaques were square‐root transformed, scaled within region, and then averaged to obtain global plaque‐tangle burden measure. Thal phase (0–5) was evaluated from seven brain regions based on the beta‐amyloid positivity using Aβ immunohistochemistry (4G8 antibody; dilution 1:9000; Covance Labs, Madison, WI).^32^ A pathological diagnosis of Alzheimer's disease, categorized as intermediate or high AD neuropathologic change (ADNC), was determined according to National Institute on Aging–Alzheimer's Association (NIA‐AA) criteria.^25^ In addition, global beta‐amyloid and paired helical filaments (PHF) ‐tau tangle densities were assessed from eight brain regions (midfrontal, superior frontal, anterior cingulate, inferior parietal, calcarine, entorhinal, and inferior temporal cortices and hippocampus) by using antibodies specific to beta‐amyloid and PHF‐tau (4G8; Biolegend, San Diego, CA; catalog # 800701; diluted at 1:9000, and AT8; Thermoscientific; catalog # MN1020; diluted at 1:3000, respectively) and digital quantification methods such as (positive pixel algorithm and Nuclear algorithm, respectively) as described previously. ^27^ Measure of hippocampal tau tangle density was used in pathway analyses.

#### 2.3.4 Hippocampal sclerosis of Aging

HS‐A was assessed in the mid‐hippocampus at the level of the lateral geniculate nucleus and was considered present if severe neuronal loss and gliosis were seen in either the CA1 or subiculum subregion of the hippocampus.^33^ Note that LATE‐NC positivity was not considered in the evaluation of HS‐A.

#### 2.3.5 Hippocampal microglia

Hippocampal microglia were assessed using immunohistochemistry with antibodies against human leukocyte antigen (HLA) class II histocompatibility antigen (clone CR3/43; DakoCytomation, Carpinteria, CA; dilution 1:100). The CA1–subiculum subregion was outlined using StereoInvestigator 8.0 software (version 2017). Within each counting frame, distinct morphological stages of microglia were counted (Figure S3) ^17^ Total microglia density for each participant was calculated by summing the counts of each stage of microglia, dividing by the outlined area, and then averaged to generate a measure for the total microglia density across all participants. ^17^

#### 2.3.6 Other age‐related brain pathologies

Other age‐related brain pathologies, including neocortical Lewy bodies (LBs),^34^ arteriolosclerosis,^34^, ^35^ atherosclerosis,^34^, ^36^ cerebral amyloid angiopathy (CAA),^20^ macroscopic infarcts,^34^ and microscopic infarcts,^37^ were evaluated using standard methods as described in the Supplement Emethod section.

### 2.4 Plasma GFAP quantification

Plasma GFAP levels were measured at the National Centralized Repository for Alzheimer's Disease and Related Dementias (NCRAD) Biomarker Assay Laboratory using standardized methods. Blood samples were centrifuged at room temperature for 10 min at 3400 rpm to separate plasma at the Rush Alzheimer's Disease Center, aliquoted, and stored at −80°C. Subsequently, plasma was sent to NCRAD and then centrifuged at 8324 × g for 5 min prior to analysis. Quantification was performed using the Quanterix Simoa Neurology 4‐Plex E Advantage Kit, following the manufacturer's instructions, except the speed of centrifugation was adjusted to accommodate different cryovial sizes. To ensure analytical quality and consistency with laboratory standards, both manufacturer‐provided quality control (QC) samples and endogenous quality controls (eQCs) were utilized. Additionally, pooled plasma reference (PPR) samples were included on each assay plate to monitor performance across analyses.^38^

### 2.5 Statistical analyses

Spearman correlations were used to examine bivariate associations of hippocampal GFAP burden with age‐at‐death, education, hippocampal microglia, amyloid‐β, and tau tangles. Analysis of variance (ANOVA) or *t‐* tests as suitable were used to compare the hippocampal GFAP measures by sex and by pathologic diagnoses (ADNC, LATE‐NC, neocortical LBs, and vascular pathologies).

We further examined these associations by performed linear regressions of hippocampal GFAP burden employing models with terms for age‐at‐death, sex, education, ADNC, LATE‐NC, neocortical LBs, and five cerebrovascular pathologies. Subsequent analyses augmented these models with terms hippocampal microglia. We also performed supporting analyses by replacing ADNC with (1) summary measure of global plaque‐tangle burden, (2) global amyloid‐beta and tau tangles as two separate terms, (3) hippocampal amyloid‐beta and tau tangles as two separate terms, and (4) Braak stage and CERAD stage as two separate terms.

Next, we employed multiple logistic regression models to examine the association of hippocampal GFAP burden with the odds of Alzheimer's dementia. All models for the Alzheimer's dementia outcome included the terms for demographic covariates (age‐at‐death, sex, and education) and hippocampal GFAP burden. We augmented the model in two ways, first by controlling: for other brain pathologies (amyloid‐β, tau tangle, LATE‐NC, neocortical LBs, arteriolosclerosis, atherosclerosis, CAA, micro‐, and macroinfarcts), and secondly by adding term for hippocampal microglia. The augmented models permit us to examine the independent association of hippocampal GFAP burden with Alzheimer's dementia above and beyond brain pathologies and hippocampal microglia.

We also employed linear mixed‐effects models of longitudinal global cognition with person‐specific random intercepts and slopes to examine the association of hippocampal GFAP burden with level and change in global cognition. The core model for the global cognition outcome included the terms for demographic covariates (age‐at‐death, sex, and education), time in years before death, and hippocampal GFAP burden. The coefficient of the time term estimates the mean annual rate of change in global cognition. We augmented the model first by controlling for other brain pathologies (amyloid‐β, tau tangle, LATE‐NC, neocortical LBs, arteriolosclerosis, atherosclerosis, CAA, micro‐, and macro infarcts), and secondly by adding the term for hippocampal microglia. A significant and negative coefficient for hippocampal GFAP term without interaction with time indicates that the GFAP burden in the hippocampus was associated with lower level of global cognition proximate to death. A significant and negative coefficient for the term of hippocampal GFAP × time suggests that GFAP burden in the hippocampus is associated with faster cognitive decline. We then repeated both core and controlled models to examine the associations of hippocampal GFAP burden with level and decline in five cognitive domains.

Finally, a path analysis was run based on the hypothesized conceptual model that the association of hippocampal GFAP with cognitive decline is partially attributable to LATE‐NC and tangles. The model was adjusted for age‐at‐death, education, and sex.

All analyses were programmed in SAS/STAT software version 9.4 (SAS Institute Inc, Cary, NC). Statistical significance was determined at nominal *α* level 0.05.

## 3 RESULTS

### 3.1 Subjects

A total of 407 participants were included in this study. The demographic, clinical, and neuropathologic characteristics of these participants are described in Table 1 and Table S1. On average, mean age at death was 92 years and mean education was 16 years. The mean of total hippocampal GFAP burden was 0.264 (SD = 0.128); the median was 0.242, and quartiles were 0.170 and 0.342.

**TABLE 1:** Characteristics of study sample (*N* = 407).

Characteristics	*N* (%) or mean (SD)
**Demographic**	
Age‐at‐death (years), mean (SD)	91.57 (5.92)
Female, no (%)	289 (71.01)
Education (years), mean (SD)	16.22 (3.65)
Non‐Latino White, no (%)	347 (85.26)
Non‐Latino Black, no (%)	43 (10.57)
Latino, no (%)	17 (4.18)
**Clinical**	
No cognitive impairment, no (%)	148 (36.36)
Mild cognitive impairment, no (%)	82 (20.15)
Alzheimer's dementia, no (%)	177 (43.49)
Global cognition, last evaluation, mean (SD)	−0.95 (1.22)
Episodic memory, last evaluation, mean (SD)	−0.80 (1.37)
Semantic memory, last evaluation, mean (SD)	−1.13 (1.40)
^a^Working memory, last evaluation, mean (SD)	−0.76 (1.17)
^a^Perceptual speed, last evaluation, mean (SD)	−1.05 (1.02)
^b^Visuospatial ability, last evaluation, mean (SD)	−0.39 (1.01)
**Neuropathologic**	
Hippocampal GFAP burden^#^, median (IQR)	0.24 (0.17‐0.34)
^c^Hippocampal microglia density^*^, median (IQR)	136.15 (2.12–403.89)
Plaque‐tangle burden summary score^^^, median (IQR)	1.06 (0.0–3.66)
Global amyloid‐β burden^#^, median (IQR)	0.88 (0.0–1.56)
Global tangle density^*^, median (IQR)	1.1 (0.02–6.26)
ADNC (Intermediate or High), no (%)	263 (64.62)
Braak NFT stages, no (%)	
None	5 (1.23)
Low (I‐II)	67 (16.46)
Intermediate (III‐IV)	216 (53.07)
High (V‐VI)	119 (29.24)
CERAD scores, no (%)	
No AD	99 (24.32)
Possible AD	34 (8.35)
Probable AD	153 (37.59)
Definite AD	121 (29.73)
LATE‐NC (Stage 2 or 3), no (%)	163 (40.05)
Hippocampal sclerosis of aging, no (%)	41 (10.07)
Neocortical Lewy bodies, no (%)	66 (16.22)
^a^Moderate‐to‐severe atherosclerosis, no (%)	107 (26.29)
Moderate‐to‐severe arteriolosclerosis, no (%)	116 (28.50)
Moderate‐to‐severe CAA, no (%)	167 (41.03)
Macroscopic infarcts, no (%)	146 (35.87)
Microscopic infarcts, no (%)	174 (42.75)

^a^Data missing for 1 participant; bData missing for 2 participants; cData missing for 23 participants.

^*^Microglia and neurofibrillary tangles were quantified as density, defined as the number of immunopositive cells per square millimeter of tissue area.

^#^GFAP and amyloid‐β were quantified as burden, defined as the percentage of tissue area positive for immunostaining.

^^^The plaque–tangle burden summary score was derived from counts of neurofibrillary tangles and neuritic plaques per square millimeter of tissue area; these measures were square root transformed, standardized, and then averaged.

Abbreviations: ADNC, Alzheimer's disease neuropathologic change; CAA, cerebral amyloid angiopathy; CERAD, Consortium to Establish a Registry for Alzheimer's Disease; GFAP, glial fibrillary acidic protein; IQR, interquartile range; LATE‐NC, limbic‐predominant age‐related TDP‐43 encephalopathy‐neuropathologic change; NFT, neurofibrillary tangles; No, number of participants; SD, standard deviation.

As expected, neurodegenerative and vascular pathologies were common, with a pathological diagnosis of AD present in 65%, LATE‐NC (stages 2 and 3) in 40%, neocortical Lewy bodies in 16%, and any type of vascular pathologies present in 84%. In bivariate analyses, hippocampal GFAP burden was positively associated with older age‐at‐death (rho = 0.256, *p* < 0.001), but not with education (rho = −0.074, *p* = 0.135) and it did not differ by sex (*p* = 0.72). Hippocampal GFAP burden was positively correlated with amyloid‐β burden (rho = 0.21, *p* < 0.001), tau tangles (rho = 0.38, *p* < 0.001), hippocampal microglia (rho = 0.522, *p* = < 0.001), ADNC (mean hippocampal GFAP burden for those with and without ADNC = 0.282 versus 0.231, *t* value −3.84, *p* < 0.001), LATE‐NC (mean hippocampal GFAP burden for those with and without LATE‐NC = 0.331 versus 0.220, t value −8.74, p < 0.001), HS‐A (mean hippocampal GFAP burden for those with and without HS‐A = 0.454 versus 0.243, *t* value −10.96, *p* < 0.001), CAA (mean hippocampal GFAP burden for those with and without CAA = 0.288 versus 0.248, *t* value −3.07, *p* = 0.002), and atherosclerosis (mean hippocampal GFAP burden for those with and without atherosclerosis = 0.298 versus 0.252, *t* value −2.95, *p* = 0.003) (Figure S4). Hippocampal GFAP burden was not associated with neocortical LBs, arteriolosclerosis, macro‐ or microscopic infarcts (all *P*s > 0.05) (Figure S4).

### 3.2 Hippocampal GFAP burden and brain pathologies

We examined the association of brain pathologies with hippocampal GFAP burden controlling for key demographic and pathology measures. ADNC and LATE‐NC were independently associated with a higher hippocampal GFAP burden (Table 2). However, the association of LATE‐NC with hippocampal GFAP burden was 3.8 times stronger than ADNC. In the same regression model, we did not observe associations of other pathologies (e.g., neocortical LBs and five vascular pathologies) with hippocampal GFAP burden. Further analyses examined the association between Lewy body disease pathology and hippocampal GFAP burden. We found similar results that nigra, limbic, and neocortical‐type LBD pathology were not associated with hippocampal GFAP burden (all three *p* values > 0.05, data not shown). Subsequent analyses examined the association of each level of ADNC and separately each stage of LATE‐NC with hippocampal GFAP. Compared to those without ADNC, only those with high ADNC had more hippocampal GFAP burden. We did not observe significant differences in hippocampal GFAP burden for individuals with low and intermediate ADNC in comparison to those with no ADNC. In contrast to ADNC, compared to people without LATE‐NC pathology, each stage of LATE‐NC (stages 1–3) was independently associated with hippocampal GFAP burden (Table S2).

**TABLE 2:** Association of brain neuropathologies with hippocampal GFAP burden.

Predictor	Hippocampal GFAP burden	
	**Model A**	**Model B**
ADNC	0.028 (0.013, 0.030)	–
Global amyloid‐β burden	–	0.001 (0.009, 0.867)
Global tangle density	–	0.029 (0.006, < 0.001)
LATE‐NC	0.095 (0.011, < 0.001)	0.084 (0.011, < 0.001)
Neocortical lewy bodies	0.011 (0.015, 0.473)	0.001 (0.015, 0.901)
Atherosclerosis	0.030 (0.014, 0.031)	0.025 (0.013, 0.067)
Arteriolosclerosis	−0.010 (0.013, 0.413)	−0.008 (0.012, 0.507)
Cerebral amyloid angiopathy	0.024 (0.012, 0.056)	0.014 (0.011, 0.209)
Macroscopic infarcts	−0.013 (0.012, 0.306)	−0.013 (0.012, 0.296)
Microscopic infarcts	−0.024 (0.012, 0.058)	−0.018 (0.011, 0.126)

*Note*: Two separate linear regression models were fitted, each with terms additionally to adjust for age‐at‐death, male sex, and education. Values in cells are estimated coefficients (Standard error, *p*‐value).

Abbreviations: GFAP, glial fibrillary acidic protein; ADNC, AD neuropathologic change.

In regard to global AD pathologic measures, global tau‐tangles but not amyloid‐β burden were associated with hippocampal GFAP burden (Table 2). Consistent findings were observed when tau tangles and amyloid‐β were replaced with Braak NFT stage and CERAD score (Table S2). Regional hippocampal TDP‐43, tangles, and amyloid‐β measures yielded similar results, with both hippocampal TDP‐43 and hippocampal tangle burden showing significant associations with hippocampal GFAP burden (Table S3). In separate analysis, a composite plaque–tangle burden measure derived from the modified Bielschowsky method was also significantly associated with hippocampal GFAP burden (estimate = 0.037; SE = 0.007; *p* < 0.001).

**FIGURE 2:**
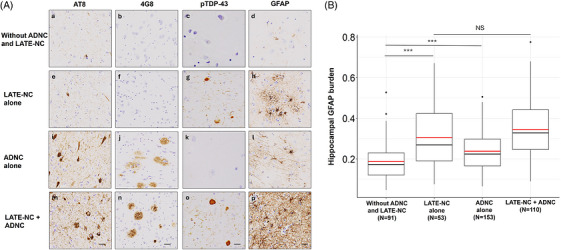
Hippocampal GFAP burden in different pathologic groups. (A) Representative images of tangles, amyloid plaques, TDP‐43 inclusions, and astrocytes in the hippocampus tissue sections stained with AT8, 4G8, pTDP‐43, and GFAP antibodies in four pathologic groups: i. Without ADNC and LATE‐NC (A–D), ii. LATE‐NC alone (E–H), iii. ADNC alone (I,L), and iv. LATE‐NC + ADNC (M–P).  (B) Distribution of hippocampal GFAP burden among those four pathologic groups. The comparisons significant (*p* < 0.05) between different groups are indicated by *** and non‐significant (*p* > 0.05) between different groups are indicated by NS. The red horizontal line in the boxplot indicates the mean level of hippocampal GFAP burden and black line indicates the median of hippocampal GFAP burden. Scale bars: 100 µm (A–P). GFAP, glial fibrillary acidic protein.

Since ADNC and LATE‐NC (with and without HS‐A) pathologies were mainly associated with hippocampal GFAP burden and both pathologies commonly co‐existed, we also compared the hippocampal GFAP burden of four different pathologic groups: no LATE‐NC and ADNC (reference group, *N* = 91), pure LATE‐NC (no ADNC, *N* = 53), pure ADNC (no LATE‐NC, *N* = 153), and LATE‐NC and ADNC (*N* = 110). **Figure** **2** illustrates hippocampal GFAP levels between the four pathologic groups. Using the ANOVA test followed by Tukey's Studentized Range test revealed that all three pathologic groups had more GFAP burden than the reference group. Furthermore, those with pure LATE‐NC exhibited greater GFAP burden than those with pure ADNC. However, no difference in GFAP burden was found between LATE‐NC groups with and without ADNC. This result may suggest the importance of hippocampal astrocytes in LATE‐NC pathophysiological process.

As we defined LATE‐NC in this study by the presence of TDP‐43 pathology (stage 2/3) with or without HS‐A, we further examined the association of hippocampal astrocytosis in LATE‐NC subjects with and without HS‐A. We divided the subjects into three groups: (a) TDP‐43 + HS‐A (*N* = 37), (b) TDP‐43 without HS‐A (*N* = 126), and (c) no TDP‐43 (reference group, *N* = 244). Compared with reference group, both TDP‐43 groups with and without HS‐A were associated with increased hippocampal GFAP burden (TDP‐43 + HS‐A: estimate = 0.234, SE = 0.018, *p* < 0.001; TDP‐43 without HS‐A: estimate = 0.0531, SE = 0.011, *p* < 0.001). However, GFAP burden was greater in the TDP‐43 group with HS‐A than the TDP‐43 group without HS‐A (adjusted mean difference = 0.181, SE = 0.019, *p* < 0.001).

Separately, we also examined the association between HS‐A and GFAP burden and found that HS‐A was significantly associated with increased hippocampal GFAP burden after adjusting for demographics AD, LBs, and vascular pathologies (estimate = 0.170, SE = 0.018, *p* < 0.001). Note that differences in the CA1–subiculum area between those with and without HS‐A were observed (mean area = 12,706,895.01 vs. 21,981,680.11 µm^2^) which may suggest that the association between HS‐A and GFAP should be interpreted with caution, as this association may be influenced by smaller hippocampal area. Furthermore, we were unable to assess the association between HS‐A and hippocampal GFAP in those without LATE‐NC as nearly all individuals with HS‐A had LATE‐NC pathology (stage 2‐3, *N* = 37/41), with only four HS‐A cases had absence of LATE‐NC pathology.

Subsequent analyses also examined the association of hippocampal microglia with hippocampal GFAP burden after controlling demographics and brain pathologies. A positive and significant association was found between microglia and GFAP (Table S4). In the same model, LATE‐NC remained associated with hippocampal GFAP burden.

### 3.3 Hippocampal GFAP burden and Alzheimer's dementia

**FIGURE 3:**
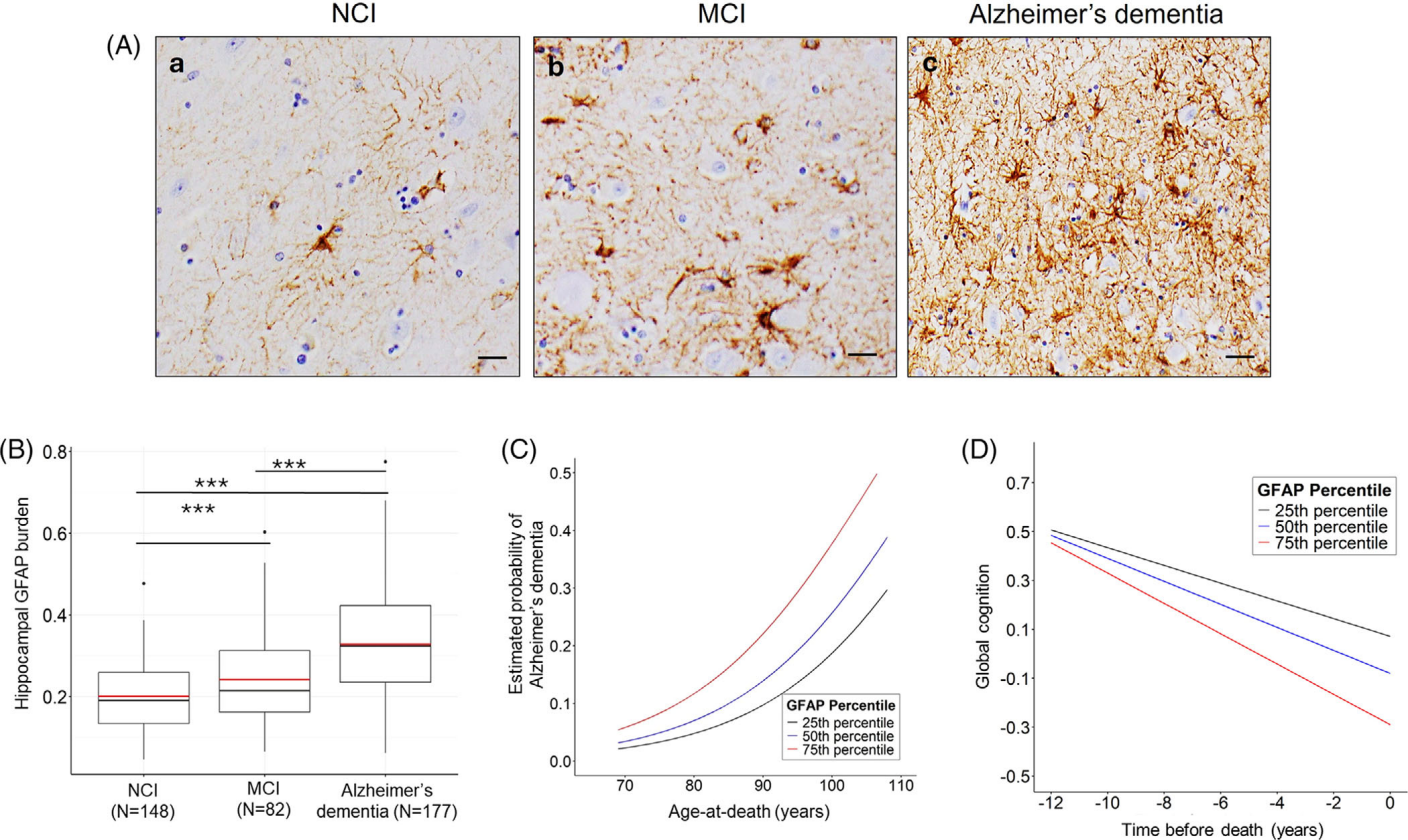
Association of hippocampal GFAP burden with Alzheimer's dementia and cognitive decline. (A) Representative images of astrocytes burden in the hippocampus tissue sections stained with GFAP antibody among three clinical groups: a. NCI, b. MCI, and c. Alzheimer's dementia. (B) Distribution of hippocampal GFAP burden in NCI, MCI, and Alzheimer's dementia groups. The significant (*p* < 0.05) comparisons between different groups are indicated by *** and the red horizontal line in the boxplot indicates the mean level of GFAP burden. (C) Estimated probability of Alzheimer's dementia and (D) Predicted path of change in global cognition for reference persons (female, age 92 year, 16 years of education, with mean of amyloid‐β and tangles and absence of LATE‐NC, arteriolosclerosis, atherosclerosis, macro‐ and microscopic infarcts, cerebral amyloid angiopathy, and neocortical Lewy bodies) with high (red line, 75th percentile), middle (blue line, 50th percentile), and low (back line, 25th percentile) hippocampal GFAP burden. Both C and D show that higher GFAP burden is associated with higher probability of Alzheimer's dementia and cognitive decline. Scale bars: 100 µm (a‐c). GFAP, glial fibrillary acidic protein; LATE‐NC, limbic‐predominant age‐related TDP‐43 encephalopathy neuropathologic changes (LATE‐NC; MCI, mild cognitive impairment; NCI, no cognitive impairment.

Next, we compared the level of hippocampal GFAP burden for those with no cognitive impairment (NCI), MCI, and Alzheimer's dementia. ANOVA test (*F* = 51.14 DFs = 2_404_, *p* = < 0.001, Figure 3A‐B) showed the groups differ; Tukey's Studentized Range test showed that those with Alzheimer's dementia diagnosis had a higher GFAP burden in comparison to those with MCI and NCI. Furthermore, those with MCI had a greater GFAP burden than those with NCI group. In the analyses adjusted for demographics, a greater GFAP burden in the hippocampus was associated with a higher odds of Alzheimer's dementia (Figure 3C). In particular, 1‐SD unit increase in hippocampal GFAP burden corresponded to a 2.6‐fold higher risk of odds of Alzheimer's dementia (odds ratio = 2.64, 95% CI = 2.03–3.43, *p* < 0.001). The association of hippocampal GFAP burden with Alzheimer's dementia attenuated by 21% but remained significant after controlling for other brain pathologies (Table 3). As expected, other brain pathologies including tangles, LATE‐NC, and neocortical LBs were also associated with Alzheimer's dementia (Table 3).

**TABLE 3:** Association of hippocampal GFAP burden with Alzheimer's dementia.

Predictor	Alzheimer's dementia
Hippocampal GFAP burden	2.06 (1.51, 2.81), *p* = < 0.001.
Global amyloid‐β burden	1.29 (0.82–2.01), *p* = 0.259.
Global tangle density	2.44 (1.75–3.40), *p* = < 0.001.
LATE‐NC	1.72 (1.01–2.97), *p* = 0.047.
Neocortical lewy bodies	3.39 (1.66–6.90), *p* < 0.001.
Atherosclerosis	1.37 (0.73–2.56), *p* = 0.317.
Arteriolosclerosis	1.58 (0.88–2.83), *p* = 0.122.
Cerebral amyloid angiopathy	0.96 (0.55–1.69), *p* = 0.911.
Macroscopic infarcts	1.35 (0.76–2.40), *p* = 0.292.
Microscopic infarcts	1.57 (0.90–2.72), *p* = 0.105.

*Note*: A single logistic regression model was applied to examine the association of hippocampal GFAP burden with risk of Alzheimer's dementia adjusted for age‐at‐death, male sex, and education. Values are expressed in odds ratio (95% CI), *p*‐value.

Abbreviations: GFAP, glial fibrillary acidic protein; LATE‐NC, limbic‐predominant age‐related TDP‐43 encephalopathy neuropathologic changes.

Because hippocampal microglia can be a potential confounder, we adjusted further for hippocampal microglia. The association of hippocampal GFAP burden with Alzheimer's dementia was again attenuated, but remained significant (odds ratio = 1.87, 95% CI = 1.32–2.63, *p* < 0.001). In the same model, hippocampal microglia were not associated with Alzheimer's dementia (*p* = 0.196).

### 3.4 Hippocampal GFAP burden and global cognition and cognitive domains

The mean follow‐up time for annual cognitive assessments was 11 (SD = 6.60) years. In the model controlling for demographics, 1‐SD unit of additional hippocampal GFAP burden was associated with an additional decline of 0.037 standard unit per year in global cognition, 0.046 unit per year in episodic memory, 0.043 unit per year in semantic memory, 0.019 unit per year in working memory, 0.020 unit in perceptual speed, and 0.012 in visuospatial ability. In further controlling for brain pathologies, the associations of hippocampal GFAP burden with decline in global cognition and four cognitive domains (i.e., episodic, semantic, and working memory and perceptual speed) were attenuated but remained significant, suggesting hippocampal GFAP burden was associated with cognitive decline above and beyond common brain pathologies (Table 4). The association of hippocampal GFAP burden and decline in visuospatial ability did not remain significant after controlling brain pathologies (Table 4). As expected, tangles, LATE‐NC, and neocortical Lewy bodies were also associated with global cognitive decline (estimate = −0.038; SE = 0.004; *p* < 0.001, estimate = −0.018; SE = 0.007; *p* = 0.011, and estimate = −0.024; SE = 0.009; *p* = 0.008, respectively). Consistent with our previous studies, amyloid‐β pathology was not associated with cognitive decline (estimate = 0.007; SE = 0.006; *p* = 0.221), when tangles were present in the same model. Figure 3D shows the rates of decline in global cognition for persons with 25^th^, 50^th^, and 75^th^ percentile of hippocampal GFAP burden, which illustrates that decline in global cognition was much faster in those with a higher GFAP burden than those with a low burden. Subsequent analyses also controlled hippocampal microglia and the association between hippocampal GFAP burden and decline in global cognition and three cognitive domains including episodic memory, semantic memory, and perceptual speed remained significant (Table 4).

**TABLE 4:** Association of hippocampal GFAP burden with level and decline in global cognition and cognitive domains.

	Level, proximate to death		Decline	
Outcome	Model A	Model B	Model A	Model B
Global cognition	−0.268 (0.049, < 0.001)	−0.218 (0.054, < 0.001)	−0.019 (0.004, < 0.001)	−0.017 (0.004, < 0.001)
Episodic memory	−0.403 (0.062, < 0.001)	−0.330 (0.067, < 0.001)	−0.026 (0.004, < 0.001)	−0.024 (0.005, < 0.001)
Semantic memory	−0.307 (0.063, < 0.001)	−0.265 (0.068, < 0.001)	−0.021 (0.005, < 0.001)	−0.019 (0.005, < 0.001)
Working memory	−0.038 (0.049, 0.435)	−0.015 (0.054, 0.784)	−0.007 (0.003, 0.026)	−0.007 (0.004, 0.085)
Perceptual speed	−0200 (0.055, < 0.001)	−0.186 (0.061, 0.002)	−0.013 (0.004, 0.003)	−0.012 (0.005, 0.013)
Visuospatial ability	−0.051 (0.050, 0.308)	−0.038 (0.056, 0.499)	−0.006 (0.004, 0.136)	−0.005 (0.004, 0.222)

*Note*: β coefficients of hippocampal GFAP burden scores from mixed‐effects models of longitudinal global and five cognitive domains outcomes. Models A was adjusted for age‐at‐death, male sex, education, brain pathologies (amyloid‐β, tangles, tangles, LATE‐NC, neocortical Lewy bodies, arteriolosclerosis, cerebral amyloid angiopathy, atherosclerosis, and macro‐ and microscopic infarcts), time expressed as years before death, and interactions of all terms with time. Model B was additionally adjusted for hippocampal microglia. Values are expressed in estimate (standard error, *p‐*value).

Abbreviations: GFAP, glial fibrillary acidic protein; LATE‐NC, limbic‐predominant age‐related TDP‐43 encephalopathy neuropathologic changes.

### 3.5 Path analyses mapping hippocampal GFAP, brain pathology, and cognitive decline

We performed path analyses to map the contribution of hippocampal GFAP to cognitive decline, focusing on the extent to which hippocampal tangles and TDP‐43 might account for this relationship as both are associated with hippocampal GFAP burden and cognitive impairment. We used person‐specific slopes of change in cognition as the outcome and hippocampal GFAP as the predictor, and regional brain pathologies such as hippocampal tangles and hippocampal TDP‐43 as mediators and we hypothesized three postulated paths: (1) a direct association of hippocampal GFAP with cognitive decline without going through pathologies, (2) an indirect association of hippocampal GFAP with cognitive decline through hippocampal tangles, and (3) an indirect association of hippocampal GFAP with cognitive decline through hippocampal TDP‐43.

**FIGURE 4:**
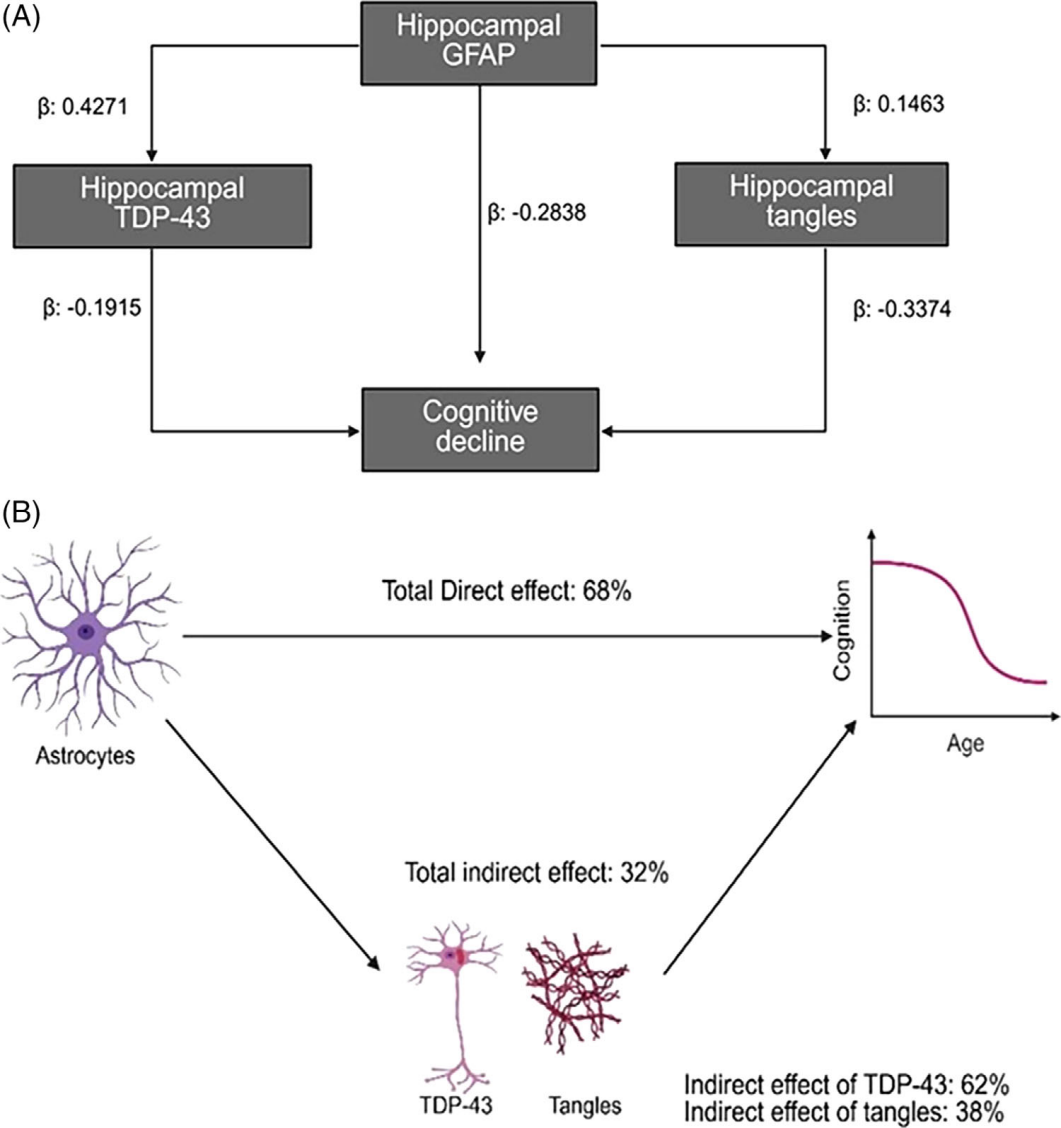
Pathway linking hippocampal GFAP, TDP‐43, tau tangles, and cognitive decline. (A) Path coefficients derived from path analysis. Each single‐headed arrow represents a hypothesized unidirectional effect of one variable on another. (B) Schematic representation of hypothesized pathway linking hippocampal GFAP, TDP‐43, and tangles with cognitive decline. GFAP, glial fibrillary acidic protein.

The total association of hippocampal GFAP burden with cognitive decline had a standardized coefficient of −0.414 (SE = 0.042, *p* < 0.001). Sixty‐eight percent was attributable to a direct association (standardized coefficient = −0.283; SE = 0.045, *p* < 0.001) and the remaining thirty‐two percent was attributable to hippocampal TDP‐43 and tangle pathologies (estimate = −0.131; SE = 0.026, *p* < 0.001). Of the in‐direct association through hippocampal TDP‐43 and tangles, 62% was attributable to hippocampal TDP‐43 and 38% was attributable to hippocampal tangle pathology (Figure 4).

### 3.6 Preliminary findings on correlation between plasma GFAP and *post mortem* GFAP

Plasma GFAP values were available on a subset of the participants (*N* = 100). The mean interval between plasma obtaining and death was 7 years (SD = 3.65). We performed preliminary analyses to investigate whether hippocampal GFAP burden was related to plasma GFAP in this subset of participants and whether the plasma GFAP and hippocampal GFAP burden relationship was differentially related to AD and LATE‐NC pathologies. Spearman correlation showed that plasma GFAP level was weak but positively correlated with *post mortem* hippocampal GFAP burden (rho = 0.24, *p *= 0.014). Interestingly, we found similar association after adjusting for the time interval between plasma collection and death (estimate = 1.08, SE = 0.46, *p* = 0.02). Additionally, in subsample of people with LATE‐NC (*N* = 44) and without LATE‐NC (*N* = 56) separately, a positive correlation between plasma GFAP and hippocampal GFAP burden was found in those with LATE‐NC (Spearman correlation rho = 0.316, *p *= 0.036) compared to those without LATE‐NC (Spearman correlation rho = 0.251, *p* = 0.089). However, this correlation was not observed among people with ADNC (*N* = 67, rho = 0.172, *p* = 0.163) and those without ADNC (*N* = 33, rho = 0.119, *p *= 0.507). These preliminary findings are intriguing and warrant further studies of brain astrocytosis and plasma GFAP markers in aging and neurodegenerative diseases.

## 4 DISCUSSION

In this clinical–pathologic study, our findings reveal that hippocampal GFAP burden is independently associated with both AD (specifically tau tangles) and LATE‐NC (with and without HS‐A) pathologies. Furthermore, the GFAP burden on the hippocampus also plays an independent role in driving late‐life cognitive decline and higher risk of clinical diagnosis of Alzheimer's dementia. Notably, approximately 32% of the association between hippocampal GFAP burden and cognitive decline was accounted for by tau tangles and LATE‐NC. Importantly, the higher GFAP burden was also associated with increased microglia density in the hippocampus, but the association of GFAP burden with cognitive decline remained independent of microglia. These results underscore the complex interplay between hippocampal astrocytes, microglia, neuropathologies, and cognitive aging and highlight a crucial role of astrocytes in both AD and LATE‐NC disease processes.

Despite growing interest of astrocytes in aging, the specific involvement of astrocytes in human *post mortem* brain pathology, specifically in the context of LATE‐NC remains poorly understood. We are aware of only one study of astrocytes in autopsied human hippocampal brain tissue. This study examined 51 autopsied participants from the University of Kentucky Alzheimer's Disease Research Center community‐based autopsy cohort. That study demonstrated a higher burden of astrocytes in those with all three pathologies (LATE‐NC + HS‐A + ADNC) compared to those with none of three pathologies.^39^ Our study demonstrates that GFAP burden in the hippocampus is associated with LATE‐NC beyond the presence of AD and other pathologies. Interestingly, the association between GFAP and LATE‐NC appears stronger than that seen with ADNC alone, pointing to a potential pathogenic role of astrocytes in LATE‐NC disease pathogenesis. Furthermore, we found that GFAP burden was higher in individuals with LATE‐NC accompanied by HS‐A, further implicating a role of astrocytes in hippocampal neurodegeneration. Overall, our findings strongly suggest that astrocyte reactivity in the hippocampus may play an important role in the pathogenesis of LATE‐NC.

We also found that tau tangle pathology is associated with hippocampal GFAP burden. The lack of association between amyloid‐β and hippocampal GFAP may reflect the regional timeline of the pathology, as amyloid deposition typically occurs in the hippocampus later than tau tangle formation. For the other brain pathologies (e.g., LBs and vascular pathologies), no associations were observed with hippocampal GFAP burden, likely reflecting the hippocampus's specific vulnerability to AD and LATE‐NC. Of note, Lewy neurites can be a prominent feature of Lewy body disease but reside mostly in CA2‐3 of the hippocampus rather than CA‐1. Furthermore, our findings may suggest a possible association between astrocytes burden and atherosclerosis (*p* = 0.06), though further study with a larger number of participants is needed for confirmation. Prior studies including our and others have shown the link between atherosclerosis and astrocytosis^40^ and hippocampal atrophy.^41^

While recent studies of plasma biomarkers for AD have shown that plasma GFAP are associated with amyloid‐β, tau pathology,^42^, ^43^ brain atrophy,^44^ and cognitive decline,^42^ the contribution of brain GFAP burden to dementia and cognitive decline remains unclear. Our results suggest that GFAP burden in the hippocampus is an important process associated with higher risks of dementia as well as a contributor to late‐life cognitive decline, even when controlling for AD, LATE‐NC, microglia, and other major brain pathologies, indicating its direct effect on dementia risk and cognitive impairment. Further these results suggest that non‐pathological factors like lifestyle factors, environmental stressors, and genetic risk alleles may influence astrocyte accumulation. Our finding of hippocampal GFAP burden with cognitive decline aligns with previous research utilizing TMT‐based proteomic data from the neocortical regions of the 386 ROSMAP participants.^45^ The strength of the current study lies in its rigorous control for confounding pathologies like AD, LATE‐NC, and others. Furthermore, our results demonstrate that the greater burden of GFAP in the hippocampus affects three key cognitive domains, particularly episodic memory, semantic memory, and perceptual speed, with the strongest association with episodic memory decline. Altogether, our study supports an important central and independent role for hippocampal astrocytes in dementia risk and cognitive decline. Future research expanding these findings may facilitate efforts to develop precision‐medicine approaches and early interventions.

As the field advances toward disease‐modifying therapies for AD and AD related dementias (AD/ADRDs), understanding underlying disease mechanisms, regional temporal heterogeneity, and structural and cognitive changes over time remain important. Our findings suggest that astrocytes are associated with cognitive deterioration through direct effects as well as through two distinct pathways: one involving LATE‐NC, and another involving neurofibrillary tangles. Importantly, LATE‐NC appears to mediate a larger portion of the astrocyte‐related impact on cognition compared to tangles. Our previous work on microglia also demonstrated relationships between microglial activation, brain pathologies, and cognitive decline.^17^ However, the interactions between microglia, astrocytes, and other glial and non‐glial cell types within the hippocampal microenvironment remain poorly understood and future studies focused on these interactions will be critical for elucidating communication pathways between microglia and astrocytes and clarifying their interactive roles in AD/ADRD‐related pathologies. While these pathways are complex, additional study is needed to clarify the sequence of pathological events, identify key molecular drivers, and uncover the mechanisms by which glial cells independently contribute to cognitive decline. Of note, a recent study using cerebrospinal fluid (CSF) from 478 individuals showed synaptic dysfunction as a potential mechanism in the association of astrocytes reactivity and cognitive deterioration.^46^

Consistent with another study,^42^ we also observed a correlation between plasma and *post mortem* hippocampal GFAP levels, supporting the notion that plasma GFAP may serve as a proxy for brain GFAP burden. Additionally, the trend toward a stronger correlation in individuals with LATE‐NC aligns with our broader finding that *post mortem* GFAP appears to mediate the pathway from TDP‐43 pathology to cognitive decline more significantly than tau tangles. Further research is needed to determine whether this relationship can be leveraged for improved in‐vivo diagnosis of LATE‐NC through biomarkers, neuropathology, and cognitive assessments.

Interestingly, plasma GFAP has been considered as a potential biomarker for disease progression in AD clinical trials.^47^, ^48^ While our study corroborates the association of hippocampal GFAP with cognitive decline and tangle pathology, it also suggests that cognitive deterioration may also stem from unrecognized TDP‐43 pathology in some trial participants particularly those with unrecognized mixed AD and LATE. Therefore, it remains important in future studies to investigate other risk factors for elevating GFAP in the blood and brain.

Our study offers several notable strengths, including well‐characterized and harmonized ROSMAPMARS community‐based longitudinal cohorts, the inclusion of a broad spectrum of AD and non‐AD brain pathologies, and the application of a quantitative approach to assess GFAP burden in the hippocampus. Despite these advantages, several limitations should be acknowledged. First, the study participants were dominated by highly educated non‐Latino White women. The underrepresentation of men, individuals with lower educational attainment, and a few participants from diverse racial and ethnic backgrounds may restrict the generalizability of our findings to broader populations. Second, the cross‐sectional nature of pathological data also limits our ability to infer causal relationships. Third, the specific role of HS‐A in relationship between GFAP and cognitive decline is not investigated in this study. This is partly due to HS‐A being defined as a binary classification, which does not reflect the full spectrum of underlying pathological changes. Future studies examining GFAP not only in relation to HS‐A status but also across varying degrees of neuronal loss may help clarify the interrelationship between astrocytosis, HS‐A, and cognitive decline. Fourth, our analysis of GFAP burden was confined to the CA1‐subiculum region of the hippocampus. Further studies investigating GFAP burden in additional brain regions, including neocortex are needed to understand the brain‐wide significance of astrocytes across LATE, AD, and other AD/ADRDs. Finally, while this study underscores the importance of hippocampal astrocytosis in brain pathology and late‐life cognitive decline, integrating structural and other neuroimaging modalities in future work could provide deeper clinical and mechanistic insights into the independent role of astrocytosis in the neurodegenerative process.

## AUTHOR CONTRIBUTIONS

Research project: Sonal Agrawal, Lei Yu, and Julie A. Schneider were involved in conception, design, and execution of the project. Manuscript: SA wrote the first draft, and Lei Yu, Sue E Leurgans, Alifiya Kapasi, Puja Agarwal, Jialiang Li, Lisa L. Barnes, Jeffrey L Dage, David A. Bennett, Patricia Boyle, and Julie A. Schneider reviewed and critiqued subsequent drafts.

## CONFLICT OF INTEREST STATEMENT

J.L.D. is an inventor on patents or patent applications assigned to Eli Lilly and Company relating to the assays, methods, reagents and / or compositions of matter for P‐tau assays and Aβ targeting therapeutics. J.L.D. has/is served/serving as a consultant or on advisory boards for Eisai, Abbvie, Genotix Biotechnologies Inc, Gates Ventures, Syndeio Biosciences, Dolby Family Ventures, Karuna Therapeutics, Alzheimer's Disease Drug Discovery Foundation, AlzPath Inc., Cognito Therapeutics, Inc., Eli Lilly and Company, Prevail Therapeutics, Neurogen Biomarking, Spear Bio, Rush University, University of Kentucky, Tymora Analytical Operations, MindImmune Therapeutics, Inc, Early is Good, and Quanterix. J.L.D. has received research support from ADx Neurosciences, Fujirebio, Roche Diagnostics and Eli Lilly and Company in the past two years. J.L.D. has received speaker fees from Eli Lilly and Company and LabCorp. J.L.D. is a founder and advisor for Monument Biosciences and Dage Scientific LLC. JLD has stock or stock options in Eli Lilly and Company, Genotix Biotechnologies, MindImmune Therapeutics Inc., AlzPath Inc., Neurogen Biomarking, and Monument Biosciences. All other authors have no conflicts of interest to declare. Author disclosures are available in the Supporting Information.

## CONSENT STATEMENT

Each cohort was separately approved by an institutional review board (IRB) of Rush University Medical Center. Each participant signed an informed consent before enrollment and a subset signed an Anatomical Gift Act to donate brain tissue for research at the time of death.

## Supporting information




**Supporting Information**: alz71613‐sup‐0001‐SuppMat.docx

**Supporting Information**: alz71613‐sup‐0002‐ICMJE.pdf

## Data Availability

The raw data can be accessed upon request through the Rush Alzheimer's Disease Center (RADC) Research Resource Sharing Hub: https://www.radc.rush.edu/
