# Supplementary material for: Hippocampal GFAP in aging: Associations with AD and LATE‐NC pathologies and cognitive decline in older adults
Source: Alzheimers Dement. 2026 Jun 17;22(6):e71613. doi: 10.1002/alz.71613 (PMC13275333; doi:10.1002/alz.71613)
Supplement: Supplementary file 1 — Supporting Information: alz71613‐sup‐0001‐SuppMat.docx [file ALZ-22-e71613-s001.docx]

**Supplementary Information**

**eMethods**

**Other neuropathologic measures**

**Neocortical Lewy bodies**— Lewy bodies were identified from seven brain regions including mid brain, amygdala, and midfrontal, middle temporal, inferior parietal, anterior cingulate, and entorhinal cortices using antibody specific to α-synuclein (pSyn, Wako Chemicals, Richmond, VA; catalog # 015-25191; diluted at 1:20,000). Neocortical Lewy bodies were coded as present if they were detected from any of the three neocortical regions (midfrontal, middle temporal, and inferior parietal cortices).^1^ As described previously ^2^, nigra, limbic, and neocortical type Lewy body disease pathology was also evaluated based on the modified McKeith criteria^2^.

**Cerebrovascular pathologies and tissue injuries**

**Atherosclerosis**—The severity of atherosclerosis was detected by gross inspection of the vertebral, basilar, anterior cerebral, middle cerebral, and posterior cerebral arteries and their proximal branches of the Circle of Willis at the base of the brain and was rated using a semiquantitative scale with 7 levels. In both descriptive and analytic analyses, a dichotomous variable graded as none-mild vs. moderate to severe was used. ^3^

**Arteriolosclerosis**— The severity of arteriolosclerosis was assessed from the vessels of the anterior basal ganglia using a semiquantitative scale from 0 (none) to 7 (occluded). A dichotomous variable none-mild vs. moderate-severe was used for the analyses.^4^

**Cerebral Amyloid Angiopathy**— Cerebral amyloid angiopathy (CAA) was assessed by measuring amyloid deposition in both meningeal and parenchymal vessels from four neocortical regions: the midfrontal, middle temporal, inferior parietal, and calcarine cortices using 4G8 antibody (1:9000; CovanceLabs, Madison, WI). The amyloid deposition in the vessels wall was scored from 0 (no deposition) to 4 (circumferential deposition over 75% of the entire region), averaged across the four regions to create a continuous summary score. A dichotomous variable none-mild vs. moderate-severe of CAA was used for the analyses.^5^

**Macroscopic and microscopic infarcts—** The presence of macroscopic infarcts were identified through gross evaluation of brain tissue and collected for the final confirmation. The presence and age of the macroscopic infarcts were confirmed through microscope evaluation. Only chronic macroscopic infarcts rated as present vs. absent were used in the analyses. Similarly, only chronic microscopic infarcts were used as identified through microscopic evaluation.^6,7^

**eFigure 1: Flowchart represents the reasons for inclusion and exclusion of the participants.**


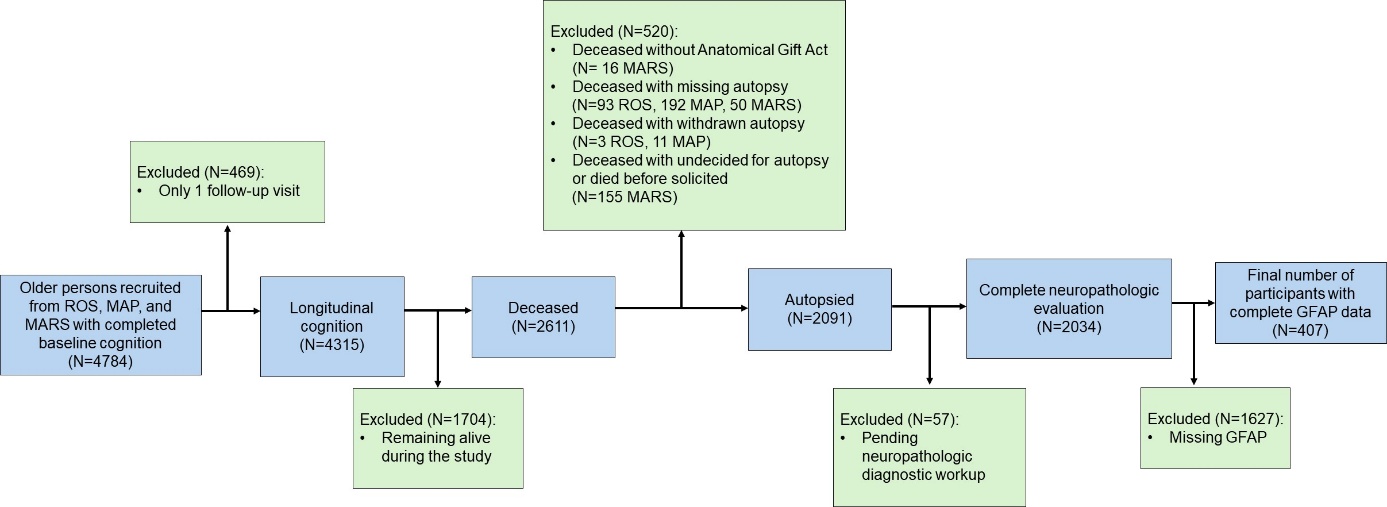


**
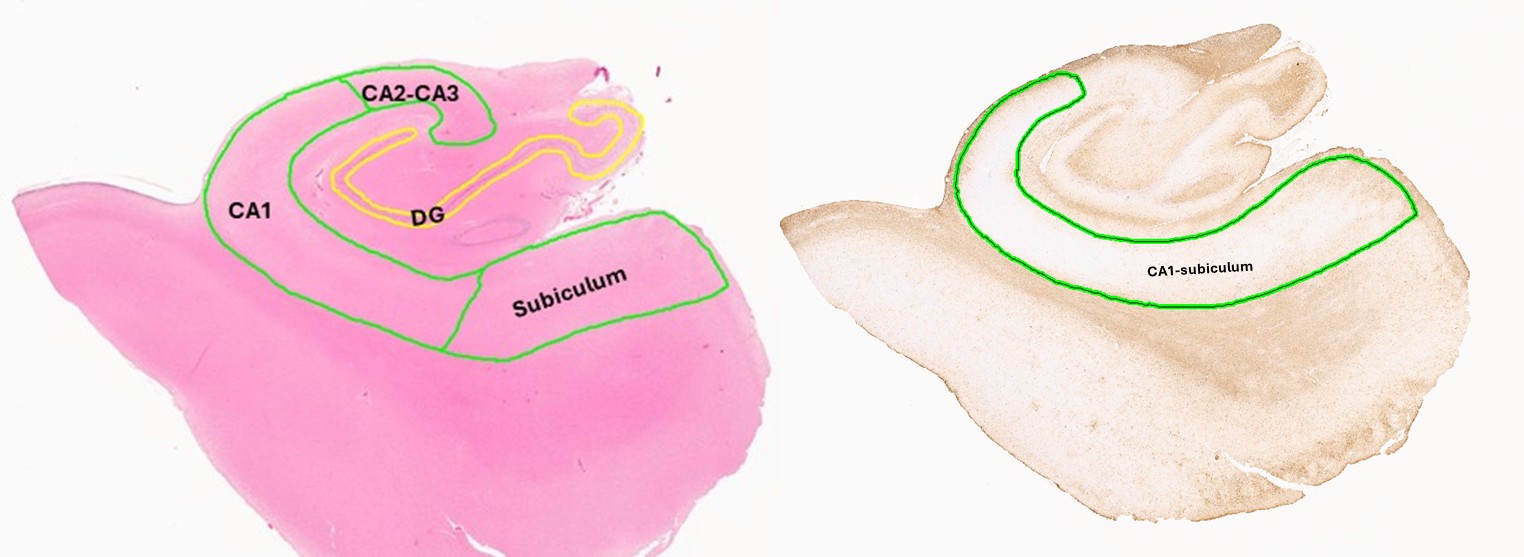
eFigure 2: Anatomical landmarks of subregions in the mid hippocampus stained with H&E (A). The CA1-subiculum subregion was annotated to quantify GFAP burden from the mid hippocampus.**

**eFigure 3: CR3-43 antibody stain of the mid hippocampus and morphologic stages of microglia.**


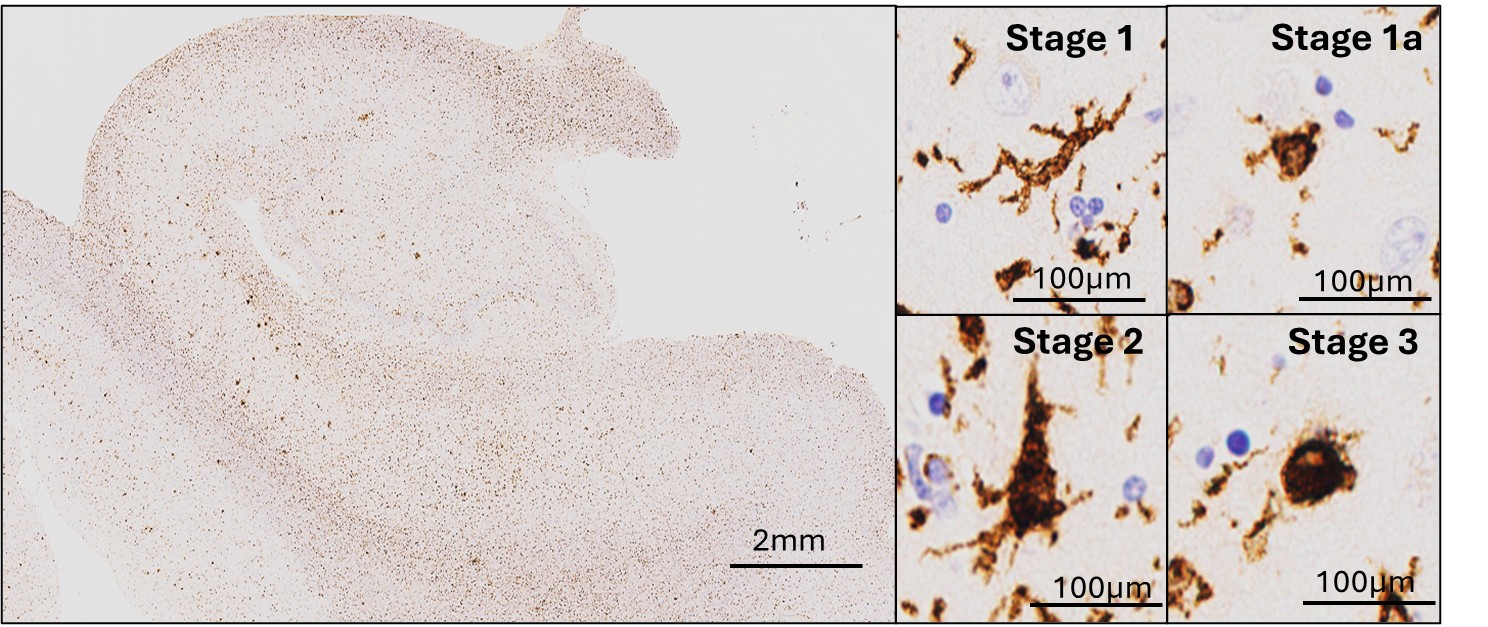


**eFigure 4: Hippocampal GFAP burden by brain neuropathologies**


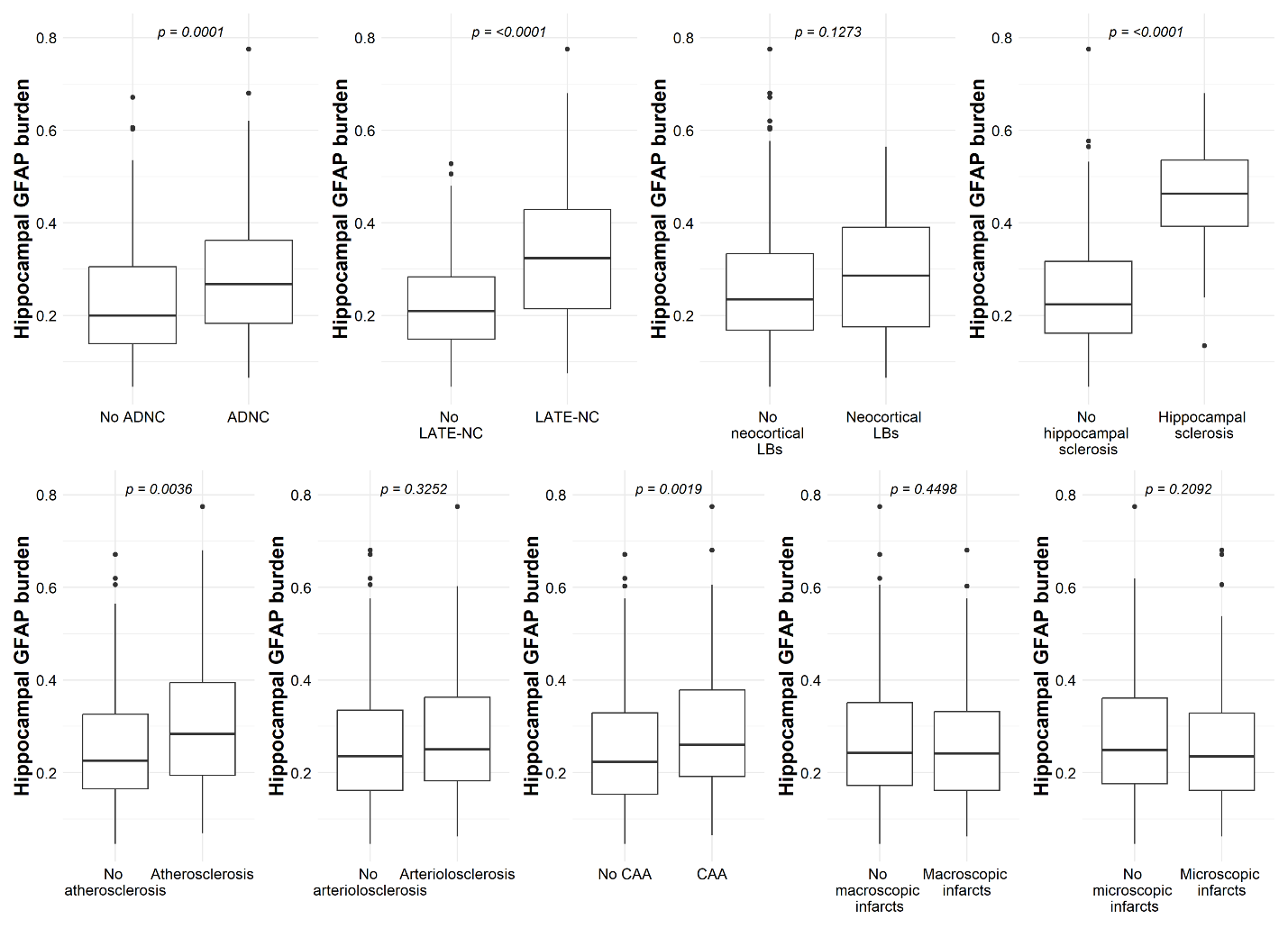


**eTable 1: Characteristics of participants by RADC cohorts**

| Characteristics | ROS (N=126) | MAP (N=251) | MARS (N=30) |
| --- | --- | --- | --- |
| Age-at-death (years), mean (SD) | 91.2 (5.1) | 92.1 (6.0) | 89.9 (6.6) |
| Female, No (%) | 91 (72.2) | 176 (70.1) | 22 (73.3) |
| Education (years), mean (SD) | 18.1 (4.1) | 15.4 (2.9) | 14.4 (3.7) |
| Alzheimer’s dementia, No (%) | 59 (46.8) | 105 (41.8) | 13 (43.3) |
| ADNC (Intermediate or High), No (%) | 94 (74.6) | 152 (60.5) | 17 (56.6) |
| Hippocampal GFAP burden, median (IQR) | 0.23 (0.15-0.32) | 0.25 (0.17-0.34) | 0.22 (0.15-0.46) |

**eTable 2: Association of ADNC and LATE-NC stages with hippocampal GFAP**

| Model | Predictor | Hippocampal GFAP burden |
| --- | --- | --- |
| A | No ADNC (N=57) | Reference |
|  | Low ADNC (N=87) | -0.014 (0.019, 0.454) |
|  | Intermediate ADNC (N=166) | 0.007 (0.017, 0.656) |
|  | High ADNC (N=97) | 0.074 (0.018, <0.001) |
| B | Braak stage | 0.024 (0.010, 0.021) |
|  | CERAD score | 0.011 (0.006, 0.075) |
| C | LATE-NC stage 0 (N=174) | Reference |
|  | LATE-NC stage 1 (N=70) | 0.039 (0.016, 0.015) |
|  | LATE-NC stage 2 (N=44) | 0.050 (0.018, 0.007) |
|  | LATE-NC stage 3 (N=119) | 0.135 (0.013, <0.001) |

Estimates derived from linear regression models. Model A and B: adjusted for age-at-death, sex, education, and other brain pathologies (LATE-NC, neocortical Lewy bodies, atherosclerosis, arteriolosclerosis, macroscopic and microscopic infarcts, and cerebral amyloid angiopathy). Model C: adjusted for age-at-death, male sex, education, and other brain pathologies (ADNC, neocortical Lewy bodies, atherosclerosis, arteriolosclerosis, macroscopic and microscopic infarcts, and cerebral amyloid angiopathy). Values in cells are estimated coefficients (standard error, P value).

**eTable 3: Association of hippocampal TDP-43, tangle, and amyloid-β with hippocampal GFAP**

| Predictor | Hippocampal GFAP burden |
| --- | --- |
| Hippocampal amyloid-β burden | 0.003 (0.003, 0.351) |
| Hippocampal tangle density | 0.009 (0.003, 0.007) |
| Hippocampal TDP-43 severity | 0.011 (0.001, <0.001) |

Estimates derived from linear regression model adjusted for age-at-death, male sex, education, and other brain pathologies (neocortical Lewy bodies, atherosclerosis, arteriolosclerosis, macroscopic and microscopic infarcts, and cerebral amyloid angiopathy). Values in cells are estimated coefficients (standard error, P value).

**eTable 4: Association between hippocampal microglia and hippocampal GFAP**

| Predictor | Hippocampal GFAP burden | | |
| --- | --- | --- | --- |
|  | Model A | Model B |  |
| Hippocampal microglia | 0.0001 (0.0001, <0.001) | 0.0008 (0.0001, <0.001) |  |
| LATE-NC |  | 0.053 (0.012, <0.001) |  |
| ADNC |  | 0.012 (0.012, 0.302) |  |

Model A was adjusted for age-at-death, male sex, and education. Model B was additionally adjusted for other brain pathologies (ADNC, LATE-NC, neocortical Lewy bodies, atherosclerosis, arteriolosclerosis, macroscopic and microscopic infarcts, and cerebral amyloid angiopathy). Values in cells are estimated coefficients (standard error, P value).

**References:**

1. Agrawal S, Yu L, Nag S, et al. The association of lewy bodies with limbic-predominant age-related TDP-43 encephalopathy neuropathologic changes and their role in cognition and alzheimer's dementia in older persons. *Acta Neuropathol Commun*. 2021;9(1):156–0.

2. Schneider JA, Arvanitakis Z, Yu L, Boyle PA, Leurgans SE, Bennett DA. Cognitive impairment, decline and fluctuations in older community-dwelling subjects with lewy bodies. *Brain*. 2012;135(Pt 10):3005–3014.

3. Oveisgharan S, Yu L, Barnes LL, et al. Association of statins with cerebral atherosclerosis and incident parkinsonism in older adults. *Neurology*. 2022;98(19):e1976–e1984. doi: 10.1212/WNL.0000000000200182.

4. Oveisgharan S, Kim N, Agrawal S, et al. Brain and spinal cord arteriolosclerosis and its associations with cerebrovascular disease risk factors in community-dwelling older adults. *Acta Neuropathol*. 2023;145(2):219–233. doi: 10.1007/s00401-022-02527-z.

5. Oveisgharan S, Yu L, Yang J, et al. Cortical gray matter proteins associated with cerebral amyloid angiopathy in community-dwelling older adults: An autopsy study. *Neurology*. 2025;105(6):e214024. doi: 10.1212/WNL.0000000000214024.

6. Schneider JA, Wilson RS, Bienias JL, Evans DA, Bennett DA. Cerebral infarctions and the likelihood of dementia from alzheimer disease pathology. *Neurology*. 2004;62(7):1148–1155.

7. Arvanitakis Z, Capuano AW, Leurgans SE, Buchman AS, Bennett DA, Schneider JA. The relationship of cerebral vessel pathology to brain microinfarcts. *Brain Pathol*. 2017;27(1):77–85.
